# Warts and Figwarts

**Published:** 1896-08

**Authors:** Olin M. Drake

**Affiliations:** Boston, Mass.


### WARTS AND FIGWARTS.

OLIN M. DRAKE, M. D., BOSTON, MASS.

[Read at a Clinical Evening of the Bœnninghausen Club, May, 1896.]

Having been for some time engaged in preparing a repertory upon warts and figwarts, the thought has occurred to me that perhaps a few personal experiences in their treatment would be an acceptable quota toward one of our monthly "feasts of reason and flow of soul." At any rate, I hope to amuse if not interest you.

Like "the peculiar people" we read of in *Punch*, warts have odd ways of their own, often disappearing as mysteriously as they originally appeared, or, in the words of the old woman, "without rhyme or reason."

As is known to you, their removal will occasionally follow all kinds of expedients, wise or otherwise, including charms, incantations, etc. My most intimate playmate during my boyhood days had two or three large seed-warts, which resisted many so-called infallible "cure-alls." They finally yielded to the following procedure: He rubbed them freely with a piece of raw beef which he stole from a butcher's cart and subsequently buried in the earth. If the beef had not been stolen the remedy would have been ineffectual, and the same failure would have attended the effort if the beef had not been put into the earth afterward. N. B.—Do not overlook these details when you try this treatment.

Having a large seed-wart myself on the second joint of the thumb of the right hand, I resorted to the same procedure, but strange as it may seem to some, I was not cured. I next tried the application of pure Nitric-acid, and later the ignition of the phosphorus of a common lucifer match over the wart, but the enemy continued "to hold the fort" with a vigor which shook my faith in such methods. But even then I began to manifest some of that obstinacy which my friends say that I possess in no small degree, and victory soon perched upon the banner of my efforts. Bits of rolled spider-webs burnt over the wart worked the miracle. After the last spark of this slow match had gone out the wart was found perfectly white, and it easily rolled out of its bed, leaving a clean and smooth surface behind. Ulceration afterward followed, painful indeed, but I was rid of my wart, though I carry to this day a cicatrix which reminds me of this incident in my youthful life. If the removal, however, had been accomplished by the homœopathic remedy, no scar would have been left, as you are all aware. He who runs may read his own inferences from this.

I have cured many cases of warts, and with the homœopathic remedy alone. No event in the earlier days of my professional life, gave me a better start in the confidence of the people of the district I was living in at the time, than the cure of a large troublesome seed-wart upon the end of the middle finger of the left hand of a master bricklayer. This wart obliged him to wear a glove during his work, for every time the wart was rubbed it would break open and bleed, and become inflamed, preventing the man from working at his trade for several days afterward. His physician ligated it, cauterized it, and even excised it, but it would sprout up again with renewed vigor, to the disgust and concern of both physician and patient. The wart vanished after he took several doses of Causticum$^{200}$.

After this cure I had many applicants for the treatment of warts, and for awhile I had quite a "rushing business" in this line. I cannot recall one single failure to cure when the patient could give me subjective indications. In the case of an isolated wart of long standing, with no, or few subjective symptoms, and the patient otherwise in good health or "symptomless," a cure would be difficult, and I fear, often impossible, but I should like to try the case before absolutely committing myself to the theory of a cure not being attainable.

When "I gird my loins" to "tackle" the enemy wart, I note down his election of domicile, the kind of "frills" he wears, or, in other words, the objective symptoms, and more especially the subjective indications. Having selected a remedy to cover all these symptoms, I give a dose of it once or twice a day for a week, and follow this with Sac-lac. for three weeks. If at the end of this time there is no improvement, and no new indications, I repeat the remedy, but in a higher potency. At the expiration of another month, should there still be no change, I seek for another remedy. Very often I have struck the bull's eye at the first shot, but not by any means invariably. If I were a better prescriber I should bring down the game each time, but alas! I am not. I must confess I have even had cases where all my efforts proved futile and the enemy came out triumphant. How I do hate to see myself thus worsted! Not only on my own account do I regret it, but for the sake of the good name of Homœopathy, the cause and success of which is so dear to myself and all Hahnemannians.

As I write there comes up to my mind the case of a "down-Easter" who had come miles to consult me. His first salutation was: "Doctor, can you do a little surgical operation for me this morning?" In reply to my inquiry as to the nature of the operation he was seeking to have performed, he stood up, unbuttoned the fly of his trousers, passed in his hooked forefinger and pulled out about six inches of a well-proportioned "life-of-man root," laid it across the palm of his left hand, and requested me to take a look at it. I had no difficulty in discovering the source of his trouble, for upon the upper surface of the glans penis was a large wart, nearly the size of a medium white bean.

Its surface was split, and each individual seed was armed with a sharp point, as hard as a porcupine's quill. I could easily imagine what a formidable implement that might prove to be, if used in the performance of the patient's marital duties. And soon the man "made no bones" of confessing that whenever he used it, he and "his woman" had a regular "cat fight."* It appeared that the difficulty between them had reached its grand climax only the day before. In the tussle she had bitten his left ear nearly off, scratched his face, and flatly declared that "never, never, so long as she had any breath in her body, would she consent to have her bowels rasped out with such a tool again." I could readily believe that that kind of "curetting" would not be likely to lead to harmonious relations between a married couple, and the wife's protests, under the circumstances, were not unreasonable. I soon convinced the man that it would be best to try internal treatment, before resorting to an operation, which I did not believe would be necessary. I administered Thuja$^{200}$ internally and touched the wart with the tincture of the same remedy. The wart soon began to shrink about its base and it continued to wither until it dropped off—about six weeks after the beginning of the treatment. If both those people do not think of me in their prayers, they are very ungrateful.

I was requested some years ago to prescribe for a little girl seven years old, whose hands were covered with a colony of warts, varying in size, numbering fifty-seven, by actual count. I prescribed Causticum$^{200}$. I did not see her again for four months, when the warts had increased to seventy-five. I then gave her Dulcamara$^{200}$, and in six weeks I repeated the same remedy in the 3,000th potency. Two months later the warts were nearly all gone, but I gave one more dose of Dulc. in the same potency as the last, when the remaining ones soon afterward disappeared. Years later I saw this same girl and she had had no return of the warts.

---

* The term used by this farmer—"cat-fight"—was not inappropriate, for the male cat's organ has rough points which makes its withdrawal from the female genitals very painful, and hence the screaming and spitting of his consort.

Many years ago I prescribed for a Shetland pony, upon and around whose vulva was a mass of figwarts which would have filled a quart measure I verily believe. I gave her Thuja internally and told her owner to apply to the parts an infusion of Thuja buds. In the course of five or six weeks the figwarts had decreased two-thirds; and I have no doubt that a complete cure would have resulted, but the pony was sold to people who took her to a distant State and I lost sight of her.

Last summer, during my summer vacation, I was consulted by a female copyist for a seed-wart located on the inner side of the little finger of the right hand, along the side of the nail. She felt stinging pains in the wart, extending toward the finger, outer side of hand, up wrist, and occasionally ascending to the arm. The wart was very sensitive to the touch. She also had another wart upon the outer side of the ring finger, just opposite the one upon the little finger, which interfered with her bringing the fingers together. Cauterizing and cutting had not done any good. Thuja$^{200}$ was given without effect, but Causticum$^{200}$ removed both warts in a few weeks.

I could relate many more similar cases, with identical results, but these will suffice for this evening.

